# Alignment and specifics of Brazilian health agencies in relation to the international premises for the implementation of digital health in primary health care: a rhetorical analysis

**DOI:** 10.3389/fsoc.2024.1303295

**Published:** 2024-02-08

**Authors:** Aguinaldo José de Araújo, Ísis de Siqueira Silva, Renan Cabral de Figueirêdo, Rayssa Horácio Lopes, Cícera Renata Diniz Vieira Silva, Osvaldo de Goes Bay Junior, Richard T. Lester, Severina Alice da Costa Uchôa

**Affiliations:** ^1^Collective Health, Federal University of Rio Grande do Norte, Natal, Brazil; ^2^Family Health, Federal University of Rio Grande do Norte, Natal, Brazil; ^3^Technical School of Health of Cajazeiras, Federal University of Campina Grande, Campina Grande, Brazil; ^4^Faculty of Health Sciences of Trairi, Federal University of Rio Grande do Norte, Natal, Brazil; ^5^Division of Infectious Diseases, Department of Medicine, University of British Columbia, Vancouver, BC, Canada; ^6^Public Health Department, Federal University of Rio Grande do Norte, Natal, Brazil

**Keywords:** telemedicine, telehealth, digital health, COVID-19, public health, primary health care

## Abstract

Digital health and sustainable development goals have had strong impacts with the COVID-19 pandemic. In Brazil, the health crisis scenario required changes in social welfare programs and policies, based on recommendations from international agencies, such as the UN and WHO. This study aims to analyze the alignment of the arguments of Brazilian and international organizations for the adoption of digital health in Primary Health Care based on the COVID-19 pandemic. This is a qualitative documentary study of the rhetorical analysis type, based on Perelman and Obrechts-Tyteca’s Theory of Argumentation. The search for documents was carried out by two independent researchers, between December 2021 and June 2022, through the websites of the World Health Organization, the Pan American Health Organization, the Brazilian Ministry of Health, and the Federal Councils of Medicine and Brazilian nursing, with the terms “digital health,” “telehealth,” “telemedicine,” “e-health,” “telehealth,” “telenursing,” “telemedicine,” and “digital health.” Twenty official documents were analyzed and identified in terms of context, authorship, authenticity, reliability, nature, and key concepts. The international and Brazilian arguments emphasize the applicability of Information and Communication Technologies (ICTs) in the health field. In logical arguments, based on the structure of reality, international agencies emphasize the overlap between health needs and the conditions for the applicability of ICTs. In Brazil, however, there was a need to regulate the digital practices of health professionals. In the international discourse, in the structuring of reality, there are illustrations of the relationship between the context of the health crisis caused by COVID-19 and the concrete conditions for the applicability of digital health; while in the Brazilian discourse, the need to strengthen an environment conducive to digital health is explicit. The Brazilian alignment in relation to the international premises is evident. Yet, there is a need, socially and economically sustainable, to strengthen the inclusion of digital health in PHC policy.

## 1 Introduction

Digital tools have been transforming health services (Pan American Health Organization (PAHO), 2020a) and contributing to ensure a healthy life and promote well-being, Sustainable Development Goal (SDG) 3 of the 2030 Agenda, proposed by the United Nations (UN) (Novillo-Ortiz et al., 2018). Digital health includes using information and communication technologies (ICTs) to solve health care problems, such as distance and poor access, especially in primary health care (PHC). Digital health was already encouraged by the World Health Organization (WHO) in middle-income countries (e.g., Brazil) and enhanced during the coronavirus disease (COVID-19) pandemic, being used in private practices, clinics, and in the Brazilian unified health system (SUS) (Caetano et al., 2020).

PHC attends to many health demands close to users and territories (World Health Organization, 2019; Rastogi, 2022). It played an important role in health promotion, prevention and education, treatment, rehabilitation, and monitoring during the COVID-19 pandemic. PHC used safe means for professionals and users to mitigate disease spread with conduct that impacted world health (Medina et al., 2018; WHO, 2020a; Silva et al., 2022).

Many countries followed the WHO guidelines on ICTs use in health. A scoping review (Silva et al., 2022) showed that phone and video calls, patient portals, cell phone applications, text messages, e-mail, electronic medical records, and social networks were highly used in health services worldwide during the pandemic. In Brazil, health professionals used mobile devices and applications to conduct appointments, safely maintain care offers, and reduce discontinuity and worsening of users under treatment (Medina et al., 2018; Celuppi et al., 2021).

The ICTs provide remote assistance services for health care users and professionals, administrative management, training, evaluation, and collaborative network research (Pan American Health Organization (PAHO), 2020b). In Brazil, the most used services are teleconsultation or teleinterconsultation, formative second opinion, telediagnosis, and tele-education (Rede APS, 2021a). A survey conducted by the PHC network of the Brazilian Association of Collective Health found that 14.5% of professionals performed teleconsultations, 16% sent prescriptions over the internet, and 42.8% used WhatsApp^Ⓡ^ for teleconsultations and patient contact. Telehealth centers also conducted several tele-education activities to fight COVID-19 (Rede APS, 2021b).

Mobile digital devices allow answering questions, patient monitoring, therapeutic assistance, interaction with professionals and health services worldwide, work continuity, self-diagnosis support, access to reliable knowledge and information, interpersonal relationships, and encouragement of healthy practices. However, their use presents some challenges that have been highlighted as obstacles to the social and economic sustainability of the use of digital health, such as information overload and management, cybersecurity (i.e., ethical and legal aspects of confidentiality of personal data), and the exclusion of people who are not digitally connected (Ramsetty and Adams, 2020; Nadav et al., 2021).

Implementing digital health in PHC needs normative regulation and adequate operation conditions to improve the adhesion of professionals and users since it is influenced by how regulations and other guiding instruments are exposed. This discussion must consider the speech adaptation, which may directly interfere with the persuasion level and adherence to the arguments (Lima, 2020), and which strategies are used to persuade heterogeneous audiences with disordered reasoning. Perelman and Olbrechts-Tyteca (2005) state that in a scenario of disorder there is a need to use multiple arguments, to adapt the speaker to the audience and order the reasoning with realistic supports that consider values, hierarchies, contexts, essences, and positions based on democratic values.

In Brazil, the public health emergency required changes in digital health regulations. During this process of changes in Brazilian legislation, national and international agencies published documents with evidence-based arguments about the importance of using and implementing digital health to expand access and optimize the provision of health services. The publication of these documents opened a space for movement between health agencies, with consonances and specificities about the challenges for the implementation of digital health care. In this sense, Brazilian and international guidelines and norms encouraged the analysis of arguments about PHC adoption of digital health in Brazil from WHO, the Pan American Health Organization (PAHO), and the Brazilian Ministry of Health (MH), Federal Council of Medicine (CFM), and Federal Council of Nursing (COFEN). Therefore, this study analyzed the arguments for adopting digital health in PHC in Brazil during and after the COVID-19 pandemic.

Thus, the rhetorical analysis of arguments from institutional documents allows the discussion about adopting digital health as a health care tool in PHC, when considering that the way in which the speaker’s deliberative speech is adapted, can directly interfere with the degree of persuasion and consequently the adhesion to the theses that are presented for convincing, since the argumentation aims at the transfer of the fundamentals and arguments of the speaker to the audience, to that, in the end, everyone comes to the same conclusion (Lima, 2020). In this context, our question is: *What arguments were used by national and international agencies for the adoption of digital health in PHC in Brazil?*

With this, the objective of this study is to analyze the alignment of arguments from Brazilian organizations to international ones for the adoption of digital health in PHC starting from COVID-19.

## 2 Methods

This qualitative document analysis used a rhetorical analysis based on the theory of argumentation of Perelman and Olbrechts-Tyteca (2005), which considered that discursive techniques and convincing arguments overlap the spoken language. Moreover, spoken language can develop using pre-selected persuasive elements as a starting point for an argument, that is the articulation of ideas, dialogs, and controversies, these being essential requirements for a pluralistic society. The rhetorical analysis consists of looking for the conditions that allow qualifying the speech about an action or a rule; it means determining what is valid and deserves to be adopted in human relationships. This approval depends on how the arguments are used to motivate the audience to make certain choices over others and, above all, to justify these, so that they become acceptable and approved by others (Lima, 2020).

The rhetorical analysis considered the analysis of techniques and arguments aimed at managers and health professionals (private auditorium) by WHO and PAHO (universal speakers), and MH, CFM, and COFEN (private speakers).

The document analysis considered the social context and health crisis of the COVID-19 pandemic to search for official publications from WHO, PAHO, MH, CFM, and COFEN regarding digital health as care, coping, and solving strategy of PHC. PHC teams in Brazil must have at least a physician and a nurse, justifying the inclusion of documents from CFM and COFEN (Brasil, 2017).

**Figure 1:**
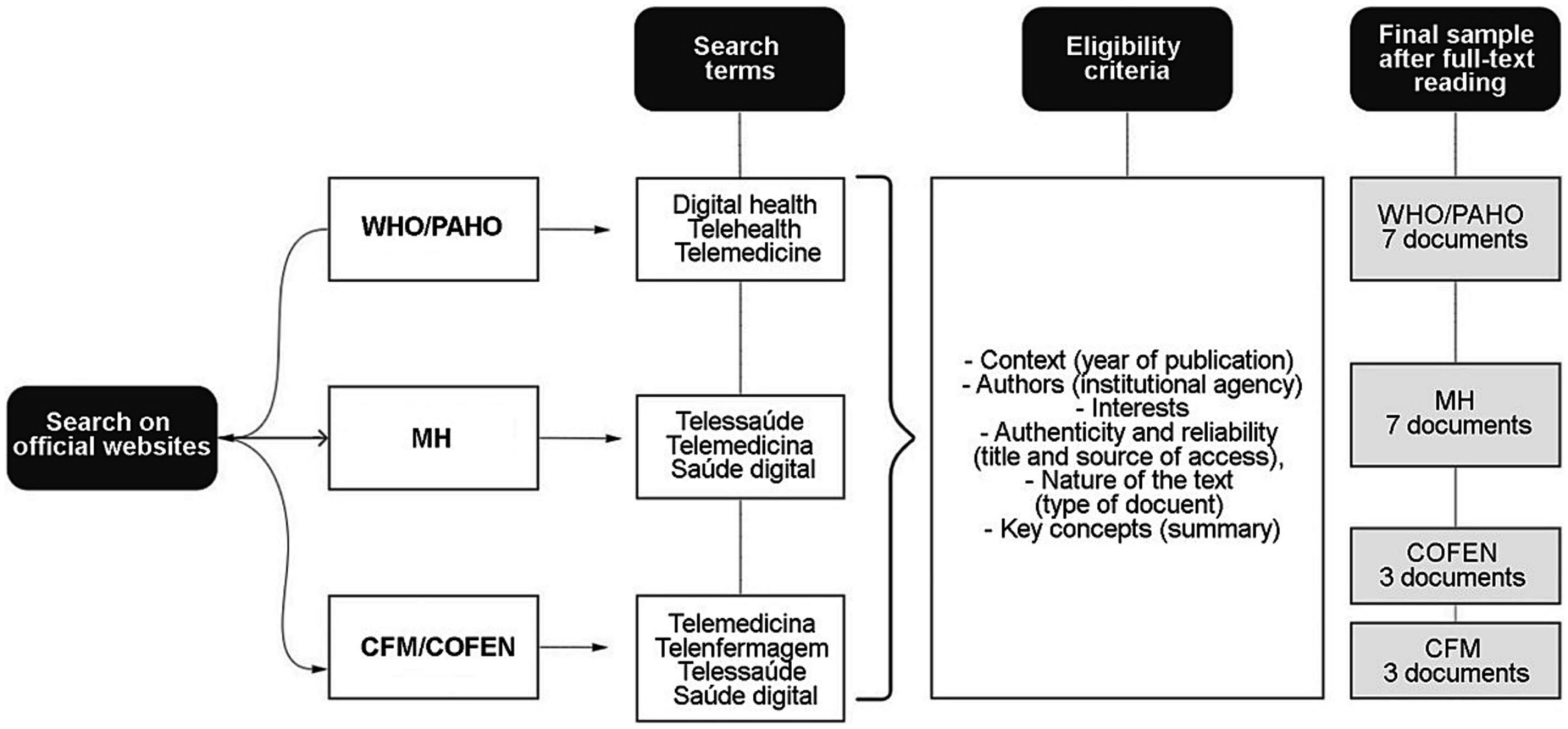
Scheme for document search.

The scheme for document search is presented in Figure 1. The documents were searched and selected between December 2021 and June 2022 on the official websites of WHO,^1^ PAHO,^2^ MH,^3^ CFM,^4^ and COFEN.^5^ The search terms individually used were “digital health,” “telehealth,” “telemedicine,” “e-health,” *“telessaúde,” “telenfermagem,” “telemedicina,”* and *“saúde digital.*” Two independent researchers (AJdA and IdSS) scanned the websites twice and found 47 documents. For inclusion in the study considered the following documents for analysis: recommendations, informative pages, guidelines, resolutions, laws, and ordinances published from March 1, 2020, to June 3, 2022. After applying the selection criteria, 27 documents were excluded for not complying with the pre-analysis of the documents recommended by Cellard (2012).

The documents met the premises proposed by Cellard (2012) before document analysis: elements of the problem or theoretical framework (theme), context (year of publication), authors (institutional agency), interests, authenticity and reliability (title and source of access), nature of the text (type of document), and key concepts (summary).

Twenty documents met the eligibility criteria, of which three were from WHO (one informative page and two guidelines), four from PAHO (four informative pages), seven from MH (one law and six ordinances), three from CFM, and three from COFEN. The critical pre-analysis of documents is presented in Chart 1 in Supplementary material.

Data were accessible to the public and directed to the universal auditorium of health systems. Data also composed the second stage of a broader study entitled “Evaluation of the quality of telemedicine in Primary Health Care in the context of COVID-19,” which was approved by the research ethics committee of the Hospital Universitário Onofre Lopes of the Federal University of Rio Grande do Norte, Brazil (registry no. 48655521.9.0000.52).

### 2.1 Contextualization and analysis of the documents

In this sense, WHO and PAHO developed instructional and informative pages (01, PAHO, 02, PAHO, 03, PAHO, 04, PAHO, 05, WHO; 06, WHO) with linking arguments that led to agreements related to the real and preferable context considering the coexistence of the pandemic with health needs. The arguments were based and justified on the structure of reality and contained strategies and guidelines for using ICTs as a digital solution to manage the new challenges in health care during the COVID-19 pandemic. In addition, the WHO launched the “Global strategy on digital health 2020–2025” (07, WHO) to help governments, ministries, and secretaries improve health using the development, adoption, and access to digital solutions. The aim was also to prevent, detect, and respond to epidemics and pandemics using infrastructure and applications that allowed countries to use data for promotion and well-being and achieve health-related sustainable development goals.

In March and April 2020, PAHO released information about teleconsultations during the COVID-19 pandemic, using the information pages “Teleconsultation during a pandemic” and “The potential of frequently used information technologies during the pandemic.” The documents encouraged diagnostic or therapeutic counseling using digital health, considering it an important strategy for public health emergencies. In addition, PAHO predicted the collapse of health services, defended the use of ICTs for information and interaction, stimulated teleconsultation to virtually help services or situations, and indicated minimum requirements for its use in PHC (01, PAHO, 02, PAHO).

On May 23, 2020, PAHO released an informative page (03, PAHO) arguing that a solid health information system may help the PHC in health promotion, prevention, and rehabilitation during the COVID-19 pandemic. In addition, the focus should be on responding to COVID-19 by integrating national and local systems, digital health, and ICTs to effectively identify, inform, and analyze cases and contacts, promptly search and detect cases, and identify and follow up on the population at risk. A solid health information system would maintain essential services during the COVID-19 pandemic and allow hospital discharge using teleconsultations. Besides, the hospital discharge would rely on follow-up, control, and rehabilitation monitoring, using medical records and electronic prescriptions in high-risk groups for severe COVID-19. Moreover, PAHO indicated that ICTs improve the cost-effectiveness of treatments, enabling the regular and uninterrupted operation of essential services.

On August 11, 2020, PAHO published the document “Digital health: a strategy to maintain health care for people living with non-communicable diseases during COVID-19” (04, PAHO), containing the three types of linking arguments. The document evidenced, with examples, how digital health tools may be applied in PHC for people with non-communicable diseases (e.g., cardiovascular or respiratory diseases, cancer, diabetes, smoking) to encourage the continuity of care during the COVID-19 pandemic. The document also considered the relocation of health professionals to combat COVID-19 and the interruption of the offer of essential services, such as public transportation (hampering the commuting of individuals and health professionals), outpatient clinics, and appointments.

The WHO published two documents (05, WHO, 06, WHO) addressing the importance of digital solutions for proximity and contact tracing. With universal writing and based on the structure of reality, the WHO stated that member States could use digital health to achieve public health goals, protect fundamental rights, and consider ethical principles (e.g., transparency, data minimization, and data storage that preserves privacy, security, accountability, and social engagement). As universal speakers, PAHO and WHO used connection arguments that influenced countries to develop digital health solutions that possibly improved and managed the quality of health care services.

Regarding the adoption of digital health in PHC by Brazilian health agencies, the initiatives were mostly conditioned to the exceptionality of the pandemic moment. The MH published Ordinance No. 467 of March 20, 2020 (08, MH), about the exceptional and temporary use of telemedicine to regulate and operationalize the SUS and supplementary and private health. This ordinance was endorsed with Law No. 13989 of April 15, 2020 (09, MH), which deliberated the use of telemedicine during a public health emergency of international importance. In addition, Ordinance No. 1768/2021 (12, MH) integrated the National Health Information and Informatics Policy (PNIIS) to assist information systems in health, support a digital transformation of the work process, improve governance in the use of information, ICT solutions, and digital health, and maintain transparency, security, and access to health information by the population.

With connection arguments based on the structure of reality, Ordinance No. 467 (08, MH) and Law No. 13989 (09, MH) stated that remote interaction could contemplate pre-clinical care, support assistance, consultation, monitoring, and diagnosis using ICTs. Also, the law determined that telemedicine should follow the usual normative and ethical standards of face-to-face care, including financial ones, since telemedicine was a medical exercise to assist, research, prevent disease and injury, and promote health.

Law No. 13989 (09, MH) encouraged the expansion of digital health along with the CFM, which regulated the emission of digital medical documents and established an integrated system to collect them with Resolutions No. 2299 (16, CFM) and 2,296 (17, CFM). These resolutions guided the medical activity during the validity of Law No. 13989, aiming to improve communication between the CFM and professionals and data security and standardize the emission of digital medical documents.

COFEN standardized telenursing during the pandemic by Resolution No. 634/2020 of March 26, 2020 (15, COFEN). Supported by arguments based on the structure of reality, the decision of the autarchy aimed to regulate population access to nursing consultations and minimize the risks of COVID-19 transmission.

On February 3, 2022, COFEN published Resolution No. 689/2022 (18, COFEN), which regulated electronic prescriptions by the nursing team. In this context, on May 4, 2022, the CFM released Resolution No. 2314/2022 (19, CFM), regulating telemedicine for physicians in Brazil. The resolution resulted from a debate with medical entities and specialists and regulated digital health regardless of the health crisis, replacing CFM Resolution No. 1643/2002. Furthermore, COFEN published Resolution No. 696/2022 (20, COFEN), standardizing the permanent use of telenursing. Via arguments based on the structure of reality (e.g., evidence proving the effectiveness of use and strict ethical, technical, and legal parameters), professional councils, as private speakers, aimed to adapt to the Brazilian scenario and strengthen the implementation of digital health.

On June 2, 2022, the MH published Ordinance No. 1348 (13, MS), regulating the definitive use of digital health in Brazil. Following COFEN and CFM, the MH reinforced the importance of digital health as a complementary strategy in health actions. It also launched a pilot project entitled Basic Digital Health Unit (BDHU) to encourage municipalities to adopt digital health in PHC, considering the national geographic diversity and remote municipalities with poor access to essential services. The pilot project was regulated by its ordinance, and criteria were established for adherence and funding based on PNIIS and the National Policy for Primary Care.

BDHU was established in PHC using Ordinance No. 1355 of June 3, 2022 (14, MH), aiming at remote rural municipalities. The goal was to spread digital health in basic health units to expand access, solvability, and integration of PHC services with Health Care Networks. With this ordinance, the MH showed city managers interest in financing and encouraging digital health implementation in PHC from 2020 to 2028, starting in remote municipalities and expanding to the entire Brazilian territory.

## 3 Results

The arguments for adopting digital health in PHC in Brazil and the world were identified by the serial number and author or agency, as specified in Chart 1 and shown in Chart 2 as Supplementary material.

Every arguments were divided into three groups according to Perelman and Olbrechts-Tyteca (2005): (1) quasi-logical arguments to guide reasoning based on evidence of information technologies in health; (2) arguments based on the structure of reality, applied to the relationship of coexistence of health needs and ICTs that may be used as digital solutions to improve and expand health care; and (3) the arguments that support the structure of reality, to perform generalizations from links between the health crisis, audiences, and the applicability of digital health, using examples, illustrations, and models of elucidation.

## 4 Discussion

WHO and PAHO demanded commitment from governmental organizations worldwide to face the international health crisis caused by the COVID-19 pandemic. In a structured PHC, ICT platforms connect components and establish specialized services when needed (Gudi et al., 2021).

In this sense, the arguments used by WHO and PAHO consolidate the need for countries to improve investment policies in technological resources and the adoption or development of digital platforms that enable the longitudinally of care and strengthen the link between users and health services (Gómez-Ramírez et al., 2021). Although there are barriers to the application of these technologies in places with limited resources (Bardosh et al., 2017; McCool et al., 2022), there is evidence that proves, for example, that the use of mobile telephony is significant for approaches to population health, such as support for oral health, stopping smoking cessation, sexual and reproductive health, and therapeutic adherence (Santiago-Torres et al., 2022; Sun et al., 2023).

Despite MH acting as a private speaker at a national level, the councils are responsible for regulating and standardizing the role of each professional. The role of physicians in digital health during the COVID-19 pandemic in Brazil was regulated by a CFM letter sent to MH (Brasil, 2020a). This disagreement resulted from CFM Resolution No. 2228 of February 26, 2019 (Brasil, 2019), which revoked CFM Resolution No. 2227/2018 of February 6, 2019 (Brasil, 2018), indicating telemedicine for medical services. Resolution No. 2228 also reinstated the validity of CFM Resolution No. 1643 of August 26, 2002, which disciplined telemedicine services. However, the COVID-19 pandemic hindered the regulation of the demands of digital medical services (Brasil, 2022a).

The WHO director-general stated on April 11, 2022, that the pandemic was far from over and that COVID-19 had a worldwide effect with an unpredictable behavior of genetic variations (World Health Organization, 2022a). Contrarily, on April 22, 2022, the Minister of Health of Brazil, Marcelo Antônio Cartaxo Queiroga Lopes, declared the end of the COVID-19 public health emergency of national importance. The MH statement extinguished the resolutions about the practice of digital health by physicians and nurses during the COVID-19 pandemic and led to regulations updates by the councils.

CFM and COFEN have followed the advances in digital health in recent years, updates, and recommendations from national and international health agencies (Lopes et al., 2019; World Health Organization, 2019). However, COFEN regulations on digital health could have been formalized earlier, considering that MH and WHO were already encouraging digital health (Barbosa et al., 2016; Sarti and Coelho, 2022), and telenursing was present in the country before the pandemic, focusing on guidelines and health promotion (World Health Organization, 2019).

The regulation of digital health in Brazil was sanctioned by the President of the Republic on December 27, 2022, through Law N° 14,510 (Brasil, 2022b), supported by previous legislation that regulates the civil framework for internet use, data protection, and the exercise of health professionals. However, each municipality has the autonomy to adhere to and develop digital health (Marengo et al., 2022).

The initiatives of the Brazilian government to encourage adherence to digital health in PHC were influenced by the experience of countries that have health systems with universal coverage (Brasil, 2023). Canada used the Canada Health Infoway to successfully develop interoperable electronic records in PHC (Canada Health Infoway, 2022), and the (Australian Digital Health Agency, 2022) used the My Health Record to expand electronic medical records.

The pandemic and the arguments used by WHO and PAHO (universal speakers) pressured the decisions and recommendations of national speakers, revealing consonances and paradoxes. As private national speakers, Brazilian agencies acted cautiously during the pandemic, possibly limiting the decision-making process of managers and professionals (private auditoriums). The uncertainty in the final regulation of digital health perhaps delayed the implementation of systematized strategies (e.g., safer platforms for patient-professional interaction) that required technological investment and financial resources (Garattini et al., 2021).

The digital health adoption by MH followed recommendations of WHO and PAHO, regardless of divergences between WHO and MH arguments about the end of the COVID-19 public health emergency. However, structuring digital PHC in Brazil needs effort and an agreement about the roles of the MH and state and city health departments in implementing, maintaining, qualifying, and continuously evolving digital health (Brasil, 2020b; Gehrke et al., 2023).

The strengthening of a universal system is closely linked to the quality of access to health, regardless of geographical conditions. Digital health has a lot to contribute to strengthening the organization of services and health care actions in the PHC and SUS, considering the large territorial extension and remote communities with poor access to health professionals and services (de Faria, 2020). Besides, digital health has the potential to reduce access inequalities. However, one of the main challenges is to overcome the Brazilian socioeconomic vulnerability, which demonstrates the existence of a digital divide, since 33.9 million people are not connected to the internet and another 86.6 million are unable to connect daily, according to Instituto Locomotiva and PwC consultancy (PWC - Pricewaterhousecoopers Brasil, 2022). Overcoming the challenges linked to socioeconomic inequality requires effective intersectoral public policy efforts, which reduce inequalities and contribute to a sustainable and conducive environment for the promotion of health and well-being.

The document analysis presented the evolution of national legislation on regulating digital health. The pressure caused by the COVID-19 pandemic expanded it. In 2022, digital health was definitively regulated, strengthening the arguments supporting actions to encourage and increase its use and guaranteeing a normative structure in Brazil. Although the country overcame legal fragility, some obstacles to implementing digital health still exist regarding integrating digital strategies with national health systems. Moreover, we highlight the importance of seeking interoperability and standardizing technological tools, clinical guidelines, scientific assessment systems, and research funds to describe and assess pandemic effects and the lack of data sharing with public health authorities for epidemiological surveillance (Ohannessian et al., 2020; World Health Organization, 2020b).

The process of digital transformation of health actions driven by ICTs focuses on the creation mechanisms, strategy, and organizational value structure of a universal health system, creating new opportunities and challenges for remodeling the way it operates and impacting health promotion and people’s well-being (Wen, 2011). In this sense, the consolidation of digital health in universal systems can contribute to achieving SDG 3 of the 2030 Agenda for Sustainable Development proposed by the UN. However, it is important to consider that commercial determinants and the digital society are dimensions of the social determinants of health and are closely linked to the sustainability of economic and social systems, and it is necessary to consider the interfaces of implementation, with public policies aligned to combat exclusion and health inequalities (Pan American Health Organization (PAHO)/WHO Brasil, 2018).

Study limitations included the analysis of documents not specific to the use of digital health in PHC, even though they address strategies about it. Also, the need for successive searches for documents and study deadlines may have excluded records posteriorly published that could be important to the rhetorical analysis and understanding of the national expansion of digital health. Therefore, a future analysis update is suggested to verify the arguments justifying the results and limitations of digital health in Brazilian PHC.

## 5 Conclusion

The arguments of WHO and PAHO influenced digital health implementation in PHC. Although some analyzed documents were not specifically about its use in PHC, addressed strategies were digital solutions for PHC, focusing on promotion, prevention, monitoring, and rehabilitation.

Despite the moderate position of the MH against WHO and PAHO guidelines, the COVID-19 pandemic accelerated the adoption of digital health. After MH declared the end of the public health emergency, professional councils encouraged MH to regulate the definitive use of digital health as a complementary health strategy. However, Brazil still needs to develop concrete actions on digital health as a safe, sustainable and complementary policy in PHC.

This study allowed us to ponder the implementation of digital health in PHC and highlighted the relevance of legislation ensuring ethics of professional practice and contributing to digital health adoption by managers and professionals. It underscores the urgency needed to dynamically adapt health ICTs and the regulatory framework to the current context to drive progress toward SDG 3. In addition, this study revealed dissonances between Brazil and international health agencies regarding the end of the COVID-19 pandemic. New studies must discuss the impacts of the arguments for health program adherence and policies.

Thus, we expect this rhetorical analysis may contribute to the theoretical and critical reflection of the actors responsible for decision-making to undertake actions that favor the promotion of health and the reduction of inequalities during the regulation, implementation, and improvement of devices and digital health systems supported by evidence, and that the methodology used can support the carrying out of other documentary research with argument analysis. Better professional performance and PHC quality with sustainability and strength beyond the pandemic context are also expected.

## Author contributions

AA: Conceptualization, Data curation, Formal analysis, Investigation, Methodology, Software, Supervision, Visualization, Writing – original draft, Writing – review & editing, Validation. ÍS: Data curation, Investigation, Methodology, Validation, Visualization, Writing – review & editing. RF: Data curation, Methodology, Visualization, Writing – original draft, Validation. RL: Data curation, Validation, Visualization, Writing – original draft. CS: Supervision, Validation, Visualization, Writing – original draft. OG: Validation, Visualization, Writing – original draft. RL: Validation, Visualization, Writing – original draft. SC: Conceptualization, Data curation, Supervision, Validation, Visualization, Writing – original draft.

## Footnotes

^1^https://apps.who.int/iris/

^2^https://www.paho.org/pt

^3^https://www.gov.br/saude/pt-br

^4^https://portal.cfm.org.br

^5^http://www.cofen.gov.br/

## References

[ref1] Australian Digital Health Agency Conectando a Austrália a um futuro mais saudável. Agência Australiana de Saúde Digital. (2022). Available at: https://www.digitalhealth.gov.au/

[ref2] Barbosa I. A. Silva K. C. C. D. Silva V. A. Silva M. J. P. (2016). The communication process in telenursing: integrative review. Rev Bras Enferm 69, 765–772. doi: 10.1590/0034-7167.2016690421i, PMID: 27508484

[ref3] Bardosh K. L. Murray M. Khaemba A. M. Smillie K. Lester R. (2017). Operationalizing mHealth to improve patient care: a qualitative implementation science evaluation of the WelTel texting intervention in Canada and Kenya. Glob. Health 13:87. doi: 10.1186/s12992-017-0311-z PMC5717811 29208026

[ref4] Brasil. Ministério da Saúde. Portaria n° 2.436, de 21 de setembro de 2017. Aprova a Política Nacional de Atenção Básica, estabelecendo a revisão de diretrizes para a organização da Atenção Básica, no âmbito do Sistema Único de Saúde (SUS). Brasília, DF: Ministério da Saúde (2017). Available at: https://bvsms.saude.gov.br/bvs/saudelegis/gm/2017/prt2436_22_09_2017.html

[ref5] Brasil. (2018). Resolução CFM n° 2.227/2018, de 6 de fevereiro de 2018. Available at: https://portal.cfm.org.br/images/PDF/resolucao222718.pdf (accessed July 24, 2023)

[ref6] Brasil. (2019). Resolução CFM n° 2.228, de 26 de fevereiro de 2019. Available at: https://www.in.gov.br/web/guest/materia/-/asset_publisher/Kujrw0TZC2Mb/content/id/65864894/

[ref7] Brasil. Ofício CFM n° 1756/2020 – COJUR. Brasília, 19 de março de (2020a). Available at: https://portal.cfm.org.br/images/PDF/2020_oficio_telemedicina.pdf (accessed July 24, 2022).

[ref8] Brasil. Ministério da Saúde, Secretaria-Executiva. Departamento de Informática do SUS. Estratégia de Saúde Digital para o Brasil 2020–2028 [recurso eletrônico]/Ministério da Saúde, Secretaria-Executiva, Departamento de Informática do SUS. Brasília: Ministério da Saúde (2020b).

[ref9] Brasil. (2022a). Lei N° 14.510, de 27 de dezembro de 2022. Available at: https://www.planalto.gov.br/ccivil_03/_ato2019-2022/2022/lei/l14510.htm (accessed July 11, 2023).

[ref10] Brasil. (2022b). Resolução CFM n° 1.643/2002, de 26 de agosto de 2002. Available at: https://sistemas.cfm.org.br/normas/visualizar/resolucoes/BR/2002/1643 (accessed July 21, 2023)

[ref11] Brasil. (2023). Estudo De Modelos Internacionais De Governança Em Saúde Digital. Available at: https://www.gov.br/saude/pt-br/assuntos/saude-digital/material-de-apoio/ModelosinternacionaisdeGovernanaemSadeDigital.pdf (accessed July 24, 2023).

[ref12] Caetano R. Silva A. B. Guedes A. C. C. M. Paiva C. C. N. Ribeiro G. R. Santos D. L.. (2020). Desafios e oportunidades para telessaúde em tempos da pandemia pela COVID-19: uma reflexão sobre os espaços e iniciativas no contexto brasileiro. Cadernos de Saúde Pública 36:e00088920. doi: 10.1590/0102-311X00088920 32490913

[ref13] Canada Health Infoway. Integrando a saúde digital na experiência de cuidados de saúde. (2022). Available at: https://www.infoway-inforoute.ca/en/ (accessed July 24, 2023)

[ref14] Cellard A. A análise documental. A pesquisa qualitativa:enfoques epistemológicos e metodológicos. (2012). 295–316.

[ref15] Celuppi I. C. Dos Lima G. S. Rossi E. (2021). Wazlawick RS, Dalmarco EM. Uma análise sobre o desenvolvimento de tecnologias digitais em saúde para o enfrentamento da COVID-19 no Brasil e no mundo. Cadernos de Saúde Pública 37:e00243220. doi: 10.1590/0102-311X00243220 33729283

[ref16] de Faria R. M. (2020). A territorialização da Atenção Básica à Saúde do Sistema Único de Saúde do Brasil. Ciência Saúde Coletiva 25, 4521–4530. doi: 10.1590/1413-812320202511.30662018 33175059

[ref17] Garattini L. Badinella Martini M. Mannucci P. M. (2021). Melhorando os cuidados primários na Europa além do COVID-19: da telemedicina às reformas organizacionais. Intern. Emerg. Med. 16, 255–258. doi: 10.1007/s11739-020-02559-x, PMID: 33196973 PMC7668282

[ref18] Gehrke M. A. Dos Dias P. S. Do Natividade T. S. S. De Magalhes A. C. C. Braun N. Dos Pessoa S. M. (2023). Perfil dos teleatendimentos realizados pelo núcleo telessaúde-Pará de 2018 a 2019. Rev Bras Med Fam Comunidade [Internet] 18:3364.

[ref19] Gómez-Ramírez O. Iyamu I. Ablona A. Watt S. Xu A. X. T. Chang H. J.. (2021). On the imperative of thinking through the ethical, health equity, and social justice possibilities and limits of digital technologies in public health. Can. J. Public Health 112, 412–416. doi: 10.17269/s41997-021-00487-7, PMID: 33725332 PMC7962628

[ref20] Gudi N. Konapur R. John O. Sarbadhikari S. Landry M. (2021). Telemedicine supported strengthening of primary care in WHO South East Asia region: lessons from the COVID-19 pandemic experiences. BMJ Innov. 7, 580–585. doi: 10.1136/bmjinnov-2021-000699

[ref21] Lima J. C. (2020). O papel da argumentação em processos deliberativos nas instâncias de controle social do Sistema Único de Saúde. Interface - Comunicação, Saúde. Educação 24:e190495. doi: 10.1590/Interface.190495

[ref22] Lopes M. Q. De Oliveria G. M. M. Maia L. M. (2019). Digital health, universal right, duty of the state? Arquivos Brasileiros de Cardiologia 113, 429–434. doi: 10.5935/abc.20190161 31482949 PMC6882383

[ref23] Marengo L. L. Kozyreff A. M. Moraes F. S. Maricato L. I. G. Barberato-Filho S. (2022). Tecnologias móveis em saúde: reflexões sobre desenvolvimento, aplicações, legislação e ética. Rev. Panam. Salud Publica 46:e37. doi: 10.26633/RPSP.2022.37, PMID: 35620177 PMC9128660

[ref24] McCool J. Dobson Whittaker R. Paton C. (2022). Saúde móvel (mHealth) em países de baixa e média renda. Revisão Anual de Saúde Pública 43, 525–539. doi: 10.1146/annurev-publhealth-052620-093850 34648368

[ref25] Medina M. G. Giovanella L. Bousquat A. De Mendonca M. H. M. Aquino R. (2018). Atenção primária à saúde em tempos de COVID-19: o que fazer? Cadernos de Saúde Pública 36:e00149720. doi: 10.1590/0102-311X00149720 32813791

[ref26] Nadav J. Kaihlanen A. Kujala S. Laukka E. Hilama P. Koivisto J.. (2021). How to implement digital Services in a way that They Integrate into Routine Work: qualitative interview study among health and social care professionals. J. Med. Internet Res. 23:e31668. doi: 10.2196/31668, PMID: 34855610 PMC8686404

[ref27] Novillo-Ortiz D. De Fátima Marin H. Saigí-Rubió F. (2018). The role of digital health in supporting the achievement of the sustainable development goals (SDGs). Int. J. Med. Inform. 114, 106–107. doi: 10.1016/j.ijmedinf.2018.03.011, PMID: 29602629

[ref28] Ohannessian R. Duong T. A. Odone A. (2020). Global telemedicine implementation and integration within health systems to fight the COVID-19 pandemic: a call to action. JMIR Public Health Surveill. 6:e18810. doi: 10.2196/18810, PMID: 32238336 PMC7124951

[ref29] Pan American Health Organization (PAHO). Saúde Digital: Uma Estratégia Para Manter A Assistência À Saúde De Pessoas Que Vivem Com Doenças Não Transmissíveis Durante A Pandemia De Covid-19, Departamento De Evidência E Inteligência Para Ação Em Saúde Vice-Diretoria. (2020a). www.paho.org/ish Página informativa N.11. Available at: https://iris.paho.org/bitstream/handle/10665.2/52576/OPASEIHISCOVID-19200015_por.pdf?sequence=4&isAllowed=y (accessed July 4, 2023)

[ref30] Pan American Health Organization (PAHO). A COVID-19 e o papel dos sistemas de informação e das tecnologias na atenção primária [Internet]. (2020b). Available at: https://iris.paho.org/bitstream/handle/10665.2/52206/OPASEIHISCOVID19200022_por.pdf?sequence=18&isAllowed=y

[ref31] Pan American Health Organization (PAHO)/WHO Brasil “Diálogo Estratégico para a Preparação do Documento de Referência para a Renovação da Promoção da Saúde no Contexto dos Objetivos de Desenvolvimento Sustentável. (Brasília, 6 a 8 de agosto de 2018). Brasilia: OPAS (2018).

[ref32] Perelman C Olbrechts-Tyteca L. Tratado da argumentação: a Nova Retórica. 6. ed São Paulo: Martins Fontes, (2005).

[ref33] PWC - Pricewaterhousecoopers Brasil. O abismo digital no Brasil. São Paulo, (2022).

[ref34] Ramsetty A. Adams C. (2020). Impact of the digital divide in the age of COVID-19. J. Am. Med. Inform. Assoc. 27, 1147–1148. doi: 10.1093/jamia/ocaa078, PMID: 32343813 PMC7197532

[ref35] Rastogi N. (2022). Healthcare’s new frontier: the digital front door. BMJ Innov.:132. doi: 10.1136/bmjinnov-2021-000874

[ref36] Rede APS (2021a). Rede de Pesquisa em APS Abrasco. Recomendações - Incorporação de recursos de telessaúde na APS no Brasil (2021). Available at: https://redeaps.org.br/wp-content/uploads/2021/10/DT-incorporacao-de-recursos-de-telessaude-na-APS-.pdf

[ref37] Rede APS (2021b). Rede De Pesquisa em APS. Nota técnica, Dezembro, 2021. Incorporação de recursos de telessaúde na Atenção primária no Brasil, 1–35, 2021. Available at: https://redeaps.org.br/wp-content/uploads/2022/01/NT_Telessaude.pdf.

[ref38] Santiago-Torres M. Mull K. E. Sullivan B. M. Ferketich A. K. Bricker J. B. (2022). Efficacy of an acceptance and commitment therapy-based smartphone application for helping rural populations quit smoking: results from the iCanQuit randomized trial. Prev. Med. 157:107008. doi: 10.1016/j.ypmed.2022.107008, PMID: 35257698 PMC9793445

[ref39] Sarti T. D. A. Coelho A. P. S. (2022). Incorporação de telessaúde na atenção primária à saúde no Brasil e fatores associados. Cadernos de Saúde Pública 38:PT252221. doi: 10.1590/0102-311XPT252221 35544879

[ref40] Silva C. R. D. V. Lopes R. H. De Goes Bay O. Jr. Martiniano C. S. Fuentealba-Torres M. Arcêncio R. A.. (2022). Digital health opportunities to improve primary health Care in the Context of COVID-19: scoping review. JMIR Hum. Factors 9:e35380. doi: 10.2196/35380, PMID: 35319466 PMC9159467

[ref41] Sun L. Qu M. Chen B. Li C. Fan H. Zhao Y. (2023). Effectiveness of mHealth on adherence to antiretroviral therapy in patients living with HIV: Meta-analysis of randomized controlled trials. JMIR Mhealth Uhealth 11, 11:e42799. doi: 10.2196/42799, PMID: 36689267 PMC9903184

[ref42] Wen C. L. (2011). “Telemedicina e telessaúde: inovação e sustentabilidade” in Organizadores. Gold book: inovação tecnológica em educação e saúde. eds. Mathias I. Monteiro A.

[ref43] WHO. (2020a). Digital implementation investment guide: integrating digital interventions into health programmes. Geneva: World Health Organization.

[ref44] World Health Organization World Health Organization guideline: Recommendations on digital interventions for health system strengthening. Geneva: World Health Organization; (2019). 31162915

[ref45] World Health Organization. (2022a). Director-General's opening remarks at the 11th meeting of the emergency committee for COVID-19. Available at: https://www.who.int/director-general/speeches/detail/who-director-general-s-opening-remarks-at-the-11th-meeting-of-the-emergency-committee-for-covid-19---11-april-2022 (accessed July 24 2022)

[ref46] World Health Organization (2020b). Digital health and COVID-19. Bull. World Health Organ. 98, 731–732. doi: 10.2471/BLT.20.021120, PMID: 33177768 PMC7607467

