# Supplementary material for: Alignment and specifics of Brazilian health agencies in relation to the international premises for the implementation of digital health in primary health care: a rhetorical analysis
Source: Front Sociol. 2024 Feb 8;9:1303295. doi: 10.3389/fsoc.2024.1303295 (PMC10881658; doi:10.3389/fsoc.2024.1303295)
Supplement: Supplementary file 1 [file Table_1.docx]

Supplementary Material

# Supplementary Charts

**Chart 1:** Critical pre-analysis of documents considering the five dimensions recommended by Cellard [18] (Appendix I).

| **Context** | | **Authors** | **Authenticity and reliability** | | **Nature of the text** | **Key concepts** |
| --- | --- | --- | --- | --- | --- | --- |
| No. | Year of publication | Author/Agency | Original title | Source | Type of document | Summary |
| 01 | 2020 | PAHO | Teleconsultations during a pandemic. | https://covid19-evidence.paho.org/handle/20.500.12663/913?locale-attribute=pt_BR. Last access on January 11, 2022. | Informative page | Informed how to conduct a teleconsultation during the pandemic. |
| 02 | 2020 | PAHO | The potential of frequently used information technologies during the pandemic. | https://iris.paho.org/handle/10665.2/52023#:~:text=Com%20mais%20de%203%20bilh%C3%B5es,usado%20pelas%20pessoas%2C%20governos%20e. Last access on March 12, 2022. | Informative page | Contextualized the use of information technologies focused on the need of interaction and information about COVID-19, support for self-diagnosis, teleconsultation, monitoring of symptoms, learning, training, and social interaction. |
| 03 | 2020 | PAHO | COVID-19 and the role of information systems and technologies in primary care. | https://iris.paho.org/handle/10665.2/52206. Last access on March 12, 2022. | Informative page | Argue on the importance of using information technologies in PHC and exemplified possible helpful tools to access health services and care. |
| 04 | 2020 | PAHO | Digital health: a strategy to maintain health care for people living with non-communicable diseases during COVID-19. | https://iris.paho.org/bitstream/handle/10665.2/52576/OPASEIHISCOVID-19200015_por.pdf?sequence=4&isAllowed=y. Last access on February 6, 2022. | Informative page | Exemplified models of interventions and digital health solutions to manage chronic non-communicable diseases during the COVID-19 pandemic. |
| 05 | 2020 | WHO | Ethical considerations to guide the use of digital proximity  tracking technologies for COVID-19 contact tracing | https://www.who.int/publications/i/item/WHO-2019-nCoV-Ethics_Contact_tracing_apps-2020.1 Last access on March 26, 2022. | Provisory guideline | Informed public health programs and governments that were developing or implementing digital technologies to trace COVID-19 contacts. The document covered ethical principles (i.e., technical considerations and requirements) and how to achieve equitable and proper use of digital solutions. |
| 06 | 2020 | WHO | Digital tools for COVID-19 contact tracing: annex: contact tracing in the context of COVID-19, 2 June 2020 | https://apps.who.int/iris/handle/10665/332265. Last access on March 12, 2022. | Informative page | Discussed tools for tracking outbreaks, proximity, and contacts of COVID-19 cases. |
| 07 | 2021 | WHO | Global strategy on digital health 2020-2025 | https://www.who.int/docs/default-source/documents/gs4dhdaa2a9f352b0445bafbc79ca799dce4d.pdf. Last access on January 11, 2022. | Guideline for global strategy | Aimed to strengthen healthcare systems using digital health for consumers, health care professionals, providers, and healthcare industries. Also, aimed to empower users and achieve the vision of health for all. |
|  | | | | | | |
| 08 | 2020 | MH | Ordinance No. 467, of March 20, 2020. | https://www.in.gov.br/en/web/dou/-  /portaria-n-467-de-20-de-marco-de-2020-249312996. Last access on January 11, 2022. | Ordinance | Exceptionally and temporarily ordered about telemedicine actions due to the COVID-19 epidemic. |
| 09 | 2020 | MH | Law No. 13989, of April 15, 2020. | https://www.in.gov.br/en/web/dou/-/  lei-n-13.989-de-15-de-abril-de-2020-252726328. Last access on January 11, 2022. | Law | Ordered about telemedicine during the health crisis caused by the COVID-19 pandemic. |
| 10 | 2020 | MH | Ordinance No. 526, of June 24, 2020. | https://www.in.gov.br/en/web/dou/-  /portaria-n-526-de-24-de-junho-de-2020-264666631. Last access on January 11, 2022. | Ordinance | Regulated the use of ICT for remote assistance, assistance support, appointments, monitoring and diagnosis, and outpatient clinics. |
| 11 | 2020 | MH | Ordinance No. 3632/2020, of December 21, 2020. | https://www.in.gov.br/en/web/dou/-/portaria-gm/ms-n-3.632-de-21-de-dezembro-de-2020-295516279. Last access on April 26, 2022. | Ordinance | Aimed to systematize, consolidate, and update the work conducted over the last decade, such as the PNIIS. |
| 12 | 2021 | MH | Ordinance GM/MS No. 1768/2021, of July 30, 2021. | https://www.in.gov.br/en/web/dou/-/portaria-gm/ms-n-1.768-de-30-de-julho-de-2021-335472332. Last access on April 26, 2022. | Ordinance | Instituted the PNIIS and its purpose. |
| 13 | 2022 | MH | Ordinance GM/MS No. 1348, of June 2, 2022 | https://www.in.gov.br/en/web/dou/-/portaria-gm/ms-n-1.348-de-2-de-junho-de-2022-405224759 | Ordinance | Ordered about telehealth actions and services in SUS. |
| 14 | 2022 | MH | Ordinance GM/MS No. 1355, of June 3, 2022. | https://www.in.gov.br/en/web/dou/-/portaria-gm/ms-n-1.355-de-3-de-junho-de-2022-405578115. Last access on April 26, 2022 | Ordinance | Established the Basic Digital Health Unit project in PHC and its purpose. |
|  | | | | | | |
| 15 | 2020 | COFEN | Resolution COFEN No. 634/2020, of March 26, 2020. | http://www.cofen.gov.br/resolucao-cofen-no-0634-2020_78344.html. Last access on January 11, 2022. | Resolution | Authorized and regulated "ad referendum" telenursing to combat the pandemic. |
| 16 | 2021 | CFM | Resolution CFM No. 2299, of September 30, 2021. | https://www.in.gov.br/en/web/dou/-  /resolucao-cfm-n-2.299-de-30-de-setembro-de-2021-354641952. Last access on January 11, 2022. | Resolution | Regulated, disciplined, and standardized the emission of electronic medical documents. |
| 17 | 2021 | CFM | Resolution CFM No. 2296/2021, of October 28, 2021. | https://sistemas.cfm.org.br/normas/visualizar/resolucoes/BR/2021/2296. Last access on January 11, 2022. | Resolution | Regulated the SIIM, which disciplined and standardized the emission of physical and digital documents of medical identification. |
| 18 | 2022 | COFEN | Resolution COFEN No. 689/2022, of February 3, 2022. | http://www.cofen.gov.br/wp-content/uploads/2022/02/RESOLUCAO-COFEN-N%C2%B0-0689-2022.pdf. Last access on February 23, 2022. | Resolution | Regulated electronic prescriptions by nurses. |
| 19 | 2022 | CFM | Resolution CFM No. 2314, of April 20, 2022. | https://www.in.gov.br/web/dou/-/resolucao-cfm-n-2.314-de-20-de-abril-de-2022-397602852. Last access on May 07, 2022. | Resolution | Defined and regulated telemedicine as a form of medical service mediated by electronic devices. |
| 20 | 2022 | COFEN | Resolution COFEN No. 696, of May 17, 2022. | https://www.in.gov.br/en/web/dou/-/resolucao-cofen-n-696-de-17-de-maio-de-2022-401809728. Last access on July 21, 2022. | Resolution | Ordered about the role of telenursing and its standardization. |

Source: Research data, 2022.

Legend: WHO – World Health Organization; PAHO - Pan American Health Organization; MH – Brazilian Ministry of Health; CFM – Brazilian Federal Council of Medicine; COFEN – Brazilian Federal Council of Nursing; PNIIS – National Health Information and Informatics Policy; SUS – Brazilian Unified Health System; SIIM - Integrated System of Medical Identification.

**Chart 2** - Documental analysis considering the theory of argumentation of Perelman e Obrech’ts-Tyteca.

| **Group 01 - Quasi-logical arguments:** applicability of ICTs in health. | |
| --- | --- |
| International documents | Brazilian documents |
| *“In general terms, for a teleconsultation is necessary to have an internet connection, a suitable computer with audio and video capabilities, and transmission devices.”* (01, PAHO)  *“Digital health, in particular teleconsultations, electronic records, and electronic prescriptions, has already proven beneficial in ensuring continuity of care especially when services are interrupted, as well as monitoring and evaluating interventions for non-communicable disease.”* (04, PAHO) | *“Article 2^nd^, the care […] should be conducted directly between health professionals and patients, using ICT that guarantees the integrity, privacy, security, and confidentiality of information.”* (13, MH)  *“The irrevocable advance of the use of ICT resources to produce and make information available and to offer reliable health services, for those who need it, when needed.”* (COFEN, 20) |
| **Group 02: Arguments based on the structure of reality:** succession and coexistence of health needs and ICTs that may be used as digital solutions to improve and expand health care. | |
| International documents | Brazilian documents |
| *“In the absence of appropriate planning and mitigation measures, health services may be exposed to the risk of collapse caused by a flood of inquiries that could be met by virtual means.”* (01, PAHO)  *“Over three billion people in the world are quarantined or in social isolation, and border closings and restrictions on transportation are in place; widely used information technologies, cell phones, in particular, have become the main way that people, governments, and health institutions work, interact, share information, exchange, generate knowledge, and communicate.”* (02, PAHO)  *“Because those are tools that many people used daily before the pandemic, and because many are accessible by cell phones, they are ideal for massive use. [...] The following are highlighted: internet, chatbots, information panels (dashboards), mobile applications (apps), social media, telephone numbers (call centers), virtual campuses, text messages (SMS), messaging services, video, and voice over the web, monitoring and tracking platforms, and forum Wikis.”* (02, PAHO)  *“Some of them are directly related to the reduced frequency of public transport services that makes it impossible for patients and health workers to commute, the closure of outpatient clinics and cancellation of appointments, and the redirection/deployment of medical staff to the COVID-19 response.”* (04, PAHO) | *“Considering the Public Health Emergency of National Importance due to human infection by the new coronavirus (2019-nCoV), declared by the Ordinance No. 188/GM/MS, of February 3, 2020; [...] the need to regulate and operationalize the measures to address the public health emergency of international importance provided for in article 3 of Law no. 13.979, of February 6, 2020, to reduce the circulation of people exposed to the COVID-19; [...] the content of the "Tel Aviv Declaration on Responsibilities and Ethical Rules in the Use of Telemedicine", adopted by the 51^st^ General Assembly of the World Medical Association, in Tel Aviv, Israel, in October 1999; [...] the possibility of prescription by the physician, treatment or other procedures without direct examination of the patient in urgent or emergency cases foreseen in the Code of Medical Ethics; [...]Resolution No. 1,643/2002 of the Federal Council of Medicine, which defines and regulates the provision of services using telemedicine; and [...] the official letter CFM No. 1756/2020-Cojur of March 19, 2020, which recognizes the possibility and ethics of using telemedicine, on an exceptional basis and while the measures to combat the coronavirus (COVID- 19) last […] Sole paragraph. The telemedicine actions referred to in the caput are conditioned to the situation of Public Health Emergency of National Importance, declared by the Ordinance No. 188/GM/MS, of February 3, 2020.”* (08, MH)  “*Article 2^nd^ During the crisis caused by the coronavirus (Sars-CoV-2), the use of telemedicine is authorized as an emergency.*” (09, MH)  *“Considering the need to monitor and update the table of procedures, medicines, orthoses, prostheses, and special materials of the Unified Health System (SUS procedures table); and considering the technical evaluation of the Department of Family Health and the Department of Regulation, Evaluation, and Control .”* (10, MH)  *“The need to improve access to health in underserved areas and with difficulty in specialized care to strengthen the strategies for preventive health adopted by the federative entities of the SUS.”* (13, MH)  *“Article 1^st^ […]regulate and operationalize the use of information and communication technologies in remote care, education, research, disease and injury prevention, management and promotion of citizens health.”* (13, MH)  *“Article 1^st^ The Basic Digital Health Unit pilot project - Digital UBS is hereby instituted within the scope of PHC. Sole paragraph. The pilot project will last 18 months after the city joins and covers all cities classified as remote rural municipalities, according to the typology established by the Brazilian Institute of Geography and Statistics.”* (14, MH)  *“Considered as Digital UBS, the Basic Health Unit - UBS is equipped with ICT resources, such as electronic medical records, worldwide computer network (internet), telehealth, interoperable health information systems, and other resources that serve as mechanisms to expand the solvability of PHC.”* (14, MH)  *“Considering the importance of the participation of nurses in fighting the pandemic, using consultations, clarifications, referrals, and guidance, especially in these moments of social isolation, when people need access to safe information and the possibility of care without traveling to health units. [...] Article. 1^st^ Authorize and standardize, "ad referendum" of the Federal Council of Nursing Plenary, nursing teleconsultation to combat the pandemic caused by the new coronavirus, using consultations, clarifications, referrals, and guidelines with the use of ICTs, with audiovisual resources and data that allow the exchange at a distance between the nurse and the patient, simultaneously or asynchronously.”* (15, COFEN)  *“Considering Law no. 14.063, of September 23, 2020, which provides for the use of electronic signatures in interactions with public entities, in acts of legal entities and health matters, and on software licenses developed by public entities.”* (16, CFM; 18, COFEN)  *“WHEREAS, the so-called “digital world” assumes a key role every day, the processes that allow remote care and the emission of medical documents in digital format gain importance (accentuated by the covid-19 pandemic), emphasizing the importance of ensuring the primacy of confidentiality in the physician-patient relationship, the safety of the process and medical practice based on professional ethics.”* (17, CFM)  *“Article 1^st^ Nursing professionals are responsible for fulfilling prescriptions remotely, provided by radio, landline or mobile telephones, messaging applications, e-mail, or any other means, in the following situations: I - mobile pre-hospital care and urgency and emergency, public or private; II – Electronic prescriptions, validated by digital or electronic signature.*” (18, COFEN)  *“WHEREAS, medicine, when exercised using safe technological and digital means, must aim at the benefit and the best results for the patient, the physician must evaluate whether telemedicine is the most appropriate method for the needs of the patient in that situation; […] the physician who uses telemedicine, aware of his legal responsibility, must evaluate whether the information received is qualified, within strict digital security protocols, and sufficient for the proposed purpose; [...] which determines Law no. 12.965, of April 23, 2014, that establishes the principles, guarantees, rights, and duties for the use of the internet in Brazil; [...] and what determines Law no. 13.709, of August 14, 2018, that provides for the personal data protection.”* (19, CFM)  *“Art. 1^st^ Standardize the performance of Nursing in Digital Health within the scope of the SUS, as well as in supplementary and private health.”* (COFEN, 20) |
| **Group 03: Arguments supporting the structure of reality:** the relationship between the context of the health crisis and the applicability of digital health. | |
| International documents | Brazilian documents |
| *“Teleconsultations are a safe and effective way to evaluate suspected cases and guide the diagnosis and treatment, minimizing the risk of disease transmission”* (01, PAHO)  *“Among the opportunities that these tools offer in the current context (e.g., access to reliable data and information in real-time and personalized recommendations on COVID-19), the following stand out: answer questions, follow up patients; therapeutic help; interact with health professionals and services; continue work; get a second opinion from professionals worldwide; support self-diagnosis; contribute to knowledge; access data and information; continue to learn; participate in social networks; maintain interpersonal relationships; verify the veracity of information; and practice prevention.”* (PAHO, 02)  *“To facilitate the effective execution of the three core functions of PHC during the pandemic, it is essential to have solid health information systems: care focused on the response to COVID-19; maintain continuity of essential services during community transmission of COVID-19; and enable discharge from hospital care not associated with COVID-19. [...] for their relevance during the pandemic in strengthening PHC, we highlight computerized disease records; provider data logging applications; applications for interaction with patients; electronic medical records; patient portals; systems for electronic prescriptions; telehealth tools; and tools for getting a second medical opinion.”* (03, PAHO)  *“ICTs increase equity in access to opportune medical care, which facilitates the assessment, diagnosis, and management of suspected and positive cases safely and effectively, minimizing the risk of transmission. As for managing health services, ICTs may help improve the cost-effectiveness of treatments and enable the regular and uninterrupted operation of key clinical services, both in preparation for and during the pandemic.”* (03, PAHO)  *“The area of digital health is expanding rapidly, and a great potential exists to apply these strategies and approaches to the provision of care for persons living with non-communicable diseases, especially when health services are disrupted, as observed during the COVID-19 pandemic.”* (04, PAHO)  *“Member States may achieve their public health objectives while protecting fundamental rights, such as privacy, simultaneously. Moreover, laws and human rights instruments provide for the use of personal data that is in the public interest, while also preventing unnecessary intrusions or commercial exploitation.”* (05, WHO)  *“Integration of digital tools for contact tracing needs to identify and address technical, cost carefully, and ethical issues.”* (06, WHO)  *“The global strategy on digital health aims to support and respond to the growing needs of countries to implement appropriate digital technologies following their health priorities and to make progress towards universal health coverage and the health-related Sustainable Development Goals. It also responds to the objectives of the Thirteenth General Program of Work of WHO, 2019–2023.”* (07, WHO) | *“Article. 5^th^, the provision of telemedicine services will follow the usual normative and ethical standards of face-to-face care, including concerning financial consideration for the service provided, with the public authority not being responsible for funding or paying for such activities when it is not exclusively a service provided to the SUS.”* (09, MH)  *"Article 244-B. The Brazilian National Digital Health Strategy 2020-2028 aims to implement, by 2028, the National Health Data Network to be established and recognized as the digital platform for innovation, information, and health services, for the benefit of users, citizens, patients, communities, managers, professionals, and health organizations.*  *"* (11, MH)  *“Article 1^st^. This Ordinance approves the National Health Information and Informatics Policy, in Annex XLII of Consolidation Ordinance GM/MS no. 2, of September 28, 2017.* *Article 2^nd^ Annex XLII of the Consolidation Ordinance GM/MS No. 2, of 2017, becomes effective in the form of the annex to this Ordinance." (12, MS)*  *“Considering the recognition of telehealth as a means of expanding universal and integral access to health attested by the scientific community, within the budgetary capacity of the Brazilian State.”* (13, MH)  *“Article. 14. The approved municipalities will be entitled to receive a financial incentive to support the implementation of the pilot project referred to in this Ordinance, in a single installment, transferred from the National Health Fund to the respective Municipal Health Funds after the publication of the Ordinance referred to in article 9 of this Ordinance, in the amount of BRL 20,000.00 (twenty thousand reais). Art. 15* *The approved municipalities, in addition to receiving an incentive for implementation, will be entitled to receive an additional monthly financial incentive for the Family Health Team and PHC team from the basic health unit participating in the pilot project, according to the provisions of art. 6 of this Ordinance, in the amount of BRL 700.00 (seven hundred reais).”* (14, MH).  *“Upon completion of the term of validity of the pilot project established in the caput, the Secretary of Primary Health Care and Ministry of Health will evaluate the results obtained* *to subsidize the decision on the eventual conversion of the pilot project into a national strategy.”* (14, MH)  “*Article 2^nd^*. *The practice of telehealth comprises nursing consultation, interconsultation, consulting, monitoring, health education, and reception of spontaneous demand mediated by ICT.*” (COFEN, 20) |

Source: research data, 2022.

Legend: COVID-19 – coronavirus disease; PHC – primary health care; ICT - Information and Communication Technology; SUS – Brazilian Unified Health System.

# Supplementary data

**References of analyzed documents**

Pan American Health Organization (PAHO). Teleconsulta durante uma pandemia. covid19-evidencepahoorg [Internet]. 2020 Mar 1 [cited 2022 Jul 4]; Available from: <https://covid19-evidence.paho.org/handle/20.500.12663/913?locale-attribute=pt_BR>.

Pan American Health Organization (PAHO). The potential of frequently used information technologies during the pandemic. Kit de ferramentas de transformação digital [Internet]. 2020 Apr 21 [cited 2022 Jul 4]; Available from: [https://iris.paho.org/handle/10665.2/52023#:~:text=Com%20 mais%20 de%203%20 milh%C3%B5](https://iris.paho.org/handle/10665.2/52023#:~:text=Com%20mais%20de%203%20bilh%C3%B5)

Pan American Health Organization (PAHO). COVID-19 and the role of information systems and technologies at the first level of care. Kit de ferramentas de transformação digital [Internet]. 2020 May 23 [cited 2022 Jul 4]; Available from: <https://iris.paho.org/handle/10665.2/52206>

Pan American Health Organization (PAHO). Saúde Digital: Uma Estratégia Para Manter A Assistência À Saúde De Pessoas Que Vivem Com Doenças Não Transmissíveis Durante A Pandemia De Covid-19 Departamento De Evidência E Inteligência Para Ação Em Saúde Vice-Diretoria. www.paho.org/ish Página informativa N.11 [Internet]. [cited 2022 Jul 4]; Available from: <https://iris.paho.org/bitstream/handle/10665.2/52576/OPASEIHISCOVID-19200015_por.pdf?sequence=4&isAllowed=y>

World Health Organization (WHO). Ethical considerations to guide the use of digital proximity tracking technologies for COVID-19 contact tracing [Internet]. [www.who.int](http://www.who.int). [cited 2022 Jul 4]; Available from: <https://www.who.int/publications/i/item/WHO-2019-nCoV-Ethics_Contact_tracing_apps-2020.1>

World Health Organization (WHO)‎. Digital tools for COVID-19 contact tracing: annex: contact tracing in the context of COVID-19, 2 June 2020. World Health Organization. <https://apps.who.int/iris/handle/10665/332265>. Licença: CC BY-NC-SA 3.0 IGO. [cited 2022 Jul 4]; Available from: <https://apps.who.int/iris/handle/10665/332265>

World Health Organization (WHO). Global strategy on digital health 2020-2025. Geneva: World Health Organization; 2021. Licence: CC BY-NC-SA 3.0 IGO. [cited 2022 Jul 4]; Available from: <https://www.who.int/docs/default-source/documents/gs4dhdaa2a9f352b0445bafbc79ca799dce4d.pdf>

Brasil. Ministério da Saúde. [Portaria nº 467, de 20 de março de 2020](http://legislacao.planalto.gov.br/legisla/legislacao.nsf/Viw_Identificacao/PRT%20467-2020?OpenDocument). Brasília, 2020. [cited 2022 Jul 21] Available from: <http://www.planalto.gov.br/ccivil_03/Portaria/PRT/Portaria%20n%C2%BA%20467-20-ms.htm>

Brasil. Lei [nº 13.989, de 15 de abril de 2020](http://legislacao.planalto.gov.br/legisla/legislacao.nsf/Viw_Identificacao/lei%2013.989-2020?OpenDocument). Brasília, 2020. [cited 2022 Jul 21] Available from: <http://www.planalto.gov.br/ccivil_03/_ato2019-2022/2020/lei/L13989.htm>.

Brasil. Ministério da Saúde. Portaria nº 526, de 24 de junho de 2020. [cited 2022 Jul 21] Available from: <https://www.in.gov.br/en/web/dou/-/portaria-n-526-de-24-de-junho-de-2020-264666631>

Brasil. Ministério da Saúde. Portaria GM/MS nº 3.632, de 21 de dezembro de 2020. [cited 2022 Jul 21] Available from: <https://www.in.gov.br/en/web/dou/-/portaria-gm/ms-n-3.632-de-21-de-dezembro-de-2020-295516279>

Brasil. Ministério da Saúde. Portaria GM/MS nº 1.768, de 30 de julho de 2021. [cited 2022 Jul 21] Available from: <https://www.in.gov.br/en/web/dou/-/portaria-gm/ms-n-1.768-de-30-de-julho-de-2021-335472332>

Brasil. Ministério da Saúde. Portaria GM/MS nº 1.348, de 02 de junho de 2022. [cited 2022 Jul 21] Available from: <https://in.gov.br/en/web/dou/-/portaria-gm/ms-n-1.348-de-2-de-junho-de-2022-405224759>

Brasil. Ministério da Saúde. Portaria GM/MS nº 1.355, de 3 de junho de 2022. [cited 2022 Jul 21] Available from: <https://www.cosemssp.org.br/wp-content/uploads/2022/06/PORTARIA-No-1.355-DE-3-DE-JUNHO-DE-2022-Institui-o-projeto-Unidade-Basica-de-Saude-Digital-UBS-Digital-no-ambito-da-Atencao-Primaria-a-Saude.pdf>

Brasil. Resolução COFEN nº 634, de 26 de março de 2020. [cited 2022 Jul 21] Available from: <http://www.cofen.gov.br/wp-content/uploads/2020/03/Resolu%C3%A7%C3%A3o-Cofen-n%C2%BA-634-2020.pdf>

Brasil. Resolução CFM nº 2.299, de 30 de setembro de 2021. [cited 2022 Jul 21] Available from: <https://www.in.gov.br/en/web/dou/-/resolucao-cfm-n-2.299-de-30-de-setembro-de-2021-354641952>

Brasil. Resolução CFM nº 2.296, de 5 de agosto de 2021. [cited 2022 Jul 21] Available from: <https://www.in.gov.br/en/web/dou/-/resolucao-cfm-n-2.296-de-5-de-agosto-de-2021-355516104>

Brasil. Resolução COFEN nº 689/2022, de 4 de fevereiro de 2022. [cited 2022 Jul 21] Available from: <http://www.cofen.gov.br/resolucao-cofen-no-689-2022_95819.html>

Brasil. Resolução CFM nº 2.314/2022, de 20 de abril de 2022. [cited 2022 Jul 21] Available from: <https://sistemas.cfm.org.br/normas/arquivos/resolucoes/BR/2022/2314_2022.pdf>

Brasil. Resolução COFEN nº 696, de 17 de maio de 2022. [cited 2022 Jul 21] Available from: <https://www.in.gov.br/en/web/dou/-/resolucao-cofen-n-696-de-17-de-maio-de-2022-401809728>
